# In vitro analysis and in vivo assessment of fracture complications associated with use of locking plate constructs for stabilization of caprine tibial segmental defects

**DOI:** 10.1186/s40634-023-00598-9

**Published:** 2023-04-03

**Authors:** Kristin M. Bowers, Ellis M. Wright, Lori D. Terrones, Xiaocun Sun, Rebecca Rifkin, Remi Grzeskowiak, Elizabeth Croy, Reza Seddighi, Stephanie Kleine, Chiara Hampton, Silke Hecht, Henry S. Adair, David E. Anderson, Pierre-Yves Mulon

**Affiliations:** 1 Large Animal Clinical Sciences, College of Veterinary Medicine, University of Tennessee, Knoxville, USA; 2 Small Animal Clinical Sciences, College of Veterinary Medicine, University of Tennessee, Knoxville, USA; 3 Office of Information Technology, University of Tennessee, Knoxville, USA

**Keywords:** Locking plate fixation, Caprine, Tibia, Plate length, Working length

## Abstract

**Purpose:**

Locking plate fixation of caprine tibial segmental defects is widely utilized for translational modeling of human osteopathology, and it is a useful research model in tissue engineering and orthopedic biomaterials research due to its inherent stability while maintaining unobstructed visualization of the gap defect and associated healing. However, research regarding surgical technique and long-term complications associated with this fixation method are lacking. The goal of this study was to assess the effects of surgeon-selected factors including locking plate length, plate positioning, and relative extent of tibial coverage on fixation failure, in the form of postoperative fracture.

**Methods:**

In vitro, the effect of plate length was evaluated using single cycle compressive load to failure mechanical testing of locking plate fixations of caprine tibial gap defects. In vivo, effects of plate length, positioning, and relative tibial coverage were evaluated using data from a population of goats enrolled in ongoing orthopedic research which utilized locking plate fixation of 2 cm tibial diaphyseal segmental defects to evaluate bone healing over 3, 6, 9, and 12 months.

**Results:**

In vitro, no significant differences in maximum compressive load or total strain were noted between fixations using 14 cm locking plates and 18 cm locking plates. In vivo, both plate length and tibial coverage ratio were significantly associated with postoperative fixation failure. The incidence of any cortical fracture in goats stabilized with a 14 cm plate was 57%, as compared with 3% in goats stabilized with an 18 cm plate. Craniocaudal and mediolateral angular positioning variables were not significantly associated with fixation failure. Decreasing distance between the gap defect and the proximal screw of the distal bone segment was associated with increased incidence of fracture, suggesting an effect on proximodistal positioning on overall fixation stability.

**Conclusions:**

This study emphasizes the differences between in vitro modeling and in vivo application of surgical fixation methods, and, based on the in vivo results, maximization of plate-to-tibia coverage is recommended when using locking plate fixation of the goat tibial segmental defect as a model in orthopedic research.

## Background

Bone healing is a complicated, non-linear process. Factors such as excessive bone loss, unfavorable healing environment, systemic disease, and biomechanical instability in long bone fracture fixation cases can lead to the formation of large defects with limited regenerative capability [25]. The risk of malunion or nonunion complications resulting in segmental defects pose innumerable surgical, socio-economic, and research challenges, and current standard treatment options are limited, sometimes necessitating limb amputation [20, 25]. In people, the tibial shaft is the most common site for segmental defects due to its size, relative lack of soft tissue coverage, and relatively frequent incidence of fracture [7, 25]. Thus, cutting-edge tissue engineering research has focused on development of synthetic bone graft substitutes and complementary regenerative therapies for these cases of tibial bone loss, fostering a need for suitable translational models [19, 25].

The most relevant species utilized for translational long bone segmental defect modeling are dogs, sheep, goats, and pigs. These species demonstrate physiologic and pathophysiologic similarities to humans in terms of long bone composition and healing, but models utilizing dogs and pigs have declined due to public concerns and limitations in handling and behavior [19, 20]. Goats are a preferred model for translational orthopedic studies due to their trainability, cost-effectiveness, and translational characteristics including body weight, long bone dimensions, bone mineral composition, metabolic rate, and bone remodeling rate compared to humans [19, 25]. The caprine tibial segmental defect model has been well documented in the literature, but fixation and stabilization options vary among research groups [11, 16–18]. Fracture fixation has varied based on clinical application, and reported methods for external and internal fixation include options such as intramedullary pins/nails, intramedullary interlocking nails, single bridging plates, or overlapping auto-compression plates [25, 30]. Orthopedic plate stabilization is a desirable option as it reflects the current standard of care for clinical management of fractures, does not interfere with the defect, and provides for ease of imaging assessment for evaluation of gap fillers including biomaterial scaffolds and other implants [25]. However, plate systems used in this model utilize a range of options including dynamic compression plates (DCP), limited-contact dynamic compression plates (LC-DCP), point-contact fixators (PC-Fix), locking compression plates (LCP), and locking plates (LP) [3, 34].

Appropriate plate selection is vital to the use of plate stabilization of segmental defects as these models lack any load sharing by the bone under investigation. At early stages of fixation, all loads exerted on the distal extremity are conducted along the plate, which is required to support the full range of biomechanical forces exerted in the tibia. As the bone defect heals and bony union is established, plate stresses decrease. However, if the plate reaches fatigue limit prior to bony union or if the fixation is biomechanically unsound, the construct will fail [11, 19, 34]. Avoidance of early loading and carefully monitored rehabilitation following plate fixation are common practices in human orthopedics to maximize fixation stability and reduce the risk of early fatigue, and immediate postoperative weightbearing in preclinical animal models poses a significant challenge to fixation longevity. Bridge plating osteosynthesis relies on a small degree of interfragmentary motion to stimulate callus formation, thus discouraging excessively stiff fixation [14, 34]. Conventional dynamic compression plating requires adequate plate to bone contact during screw tightening to achieve desired stability between the plate and bone surface, and potential complications such as periosteal vascular compromise, stress risers, and loss of compression in compromised bone may lead to fracture complications [8, 22, 35]. In contrast, locking compression plating relies more on the screw-plate interface for construct stability, and locking plate fixation is particularly useful in cases of bone loss or poor bone integrity [11, 32]. Biomechanically, DCPs and LC-DCPs convert axial load to shear stress whereas LCPs convert axial load to compressive force, and the stability of a locking plate construct will reflect the sum of screw torques, not merely the friction of the bone-plate interface [21]. Biomechanical comparisons of dynamic and locking plating using both ex vivo bone and in vitro composite bone substitute models have established that LCP fixation (bridging) of a segmental defect yields significantly stiffer constructs in compression and bending [1–3, 9, 13, 15]. However, results have varied regarding the torsional stiffness of LCP constructs with relatively early failure of LCP constructs under torsional testing compared to similar constructs exposed to bending or compression forces [1, 9]. Notably, Gardner et al. documented a consistent mode of failure when human radial constructs stabilized with LCPs were torsion tested in a cyclic fatigue model. The authors noted that LCP constructs typically fractured in a longitudinal pattern originating at the gap and extending along multiple screw holes [9].

Due to its stability and suitability for tissue engineering and bone biomaterial research, locking compression plate fixation has gained widespread use in bone segmental defect models [20, 25]. However, questions regarding risk factors for locking plate implant failure and possible torsional instability persist, and relatively few studies have been published assessing complications associated with tibial locking plate gap stabilization, particularly in small ruminant species [6, 11, 26]. Finite element analysis (FEA) has allowed exploration of intrinsic factors of LCP fixation including plate length, positioning of the plate on the bone, screw number, and screw placement. In a human FEA model utilizing medial placement of an LCP at the tibial diaphysis, construct rigidity increased directly with plate length, and the authors noted that clinically, the use of relatively longer plates could reduce the risk for fixation failure [5]. Biomechanical testing has corroborated this claim with multiple studies reporting a direct association between locking plate length and construct stiffness [27, 33]. The literature is more divided in regard to working length, the distance between the proximal and distal screws in the closest proximity to the fracture gap, with studies reporting that an increase in LCP working length could be associated with increased, decreased, or no change in construct stiffness in axial compressive testing [6, 10, 21, 33]. In human patients, single locking plate internal fixation applied in bridging fashion is commonly applied to comminuted distal femoral fractures [26]. Several retrospective studies have examined risk factors associated with fixation failures in vivo [12, 26, 28]. Patient factors such as age, concurrent systemic disease, diabetes, a history of smoking, and obesity were consistently associated with postoperative complications including infection and reoperation, but additional plate-specific factors of plate length (total) and plate length proximal to the fracture line were significantly associated with implant failure [26]. In retrospective analysis of fracture nonunion following LCP fixation of human distal femoral fractures, plate characteristics including number of locking screws in the proximal fragment and plate material (stainless steel) were significantly associated with nonunion but the factors of plate length, working length, and total number of screws were not [12, 24, 28, 29]. To our knowledge, these locking compression plate characteristics and the effect of plate position have not been assessed in vivo in a caprine segmental defect model. In addition, few studies report complications following use of locking plate fracture fixation in animals and few focus on translational research species such as sheep and goats [18]. Therefore, the goal of this research was to assess a large cohort of goats enrolled in an unrelated orthopedic study in which a tibial segmental defect was stabilized with bridging locking plates and to analyze plate-bone positioning factors associated with implant failure in the form of postoperative fracture complication.

## Materials and methods

### In vitro experiments

#### Specimen preparation

**Fig. 1:**
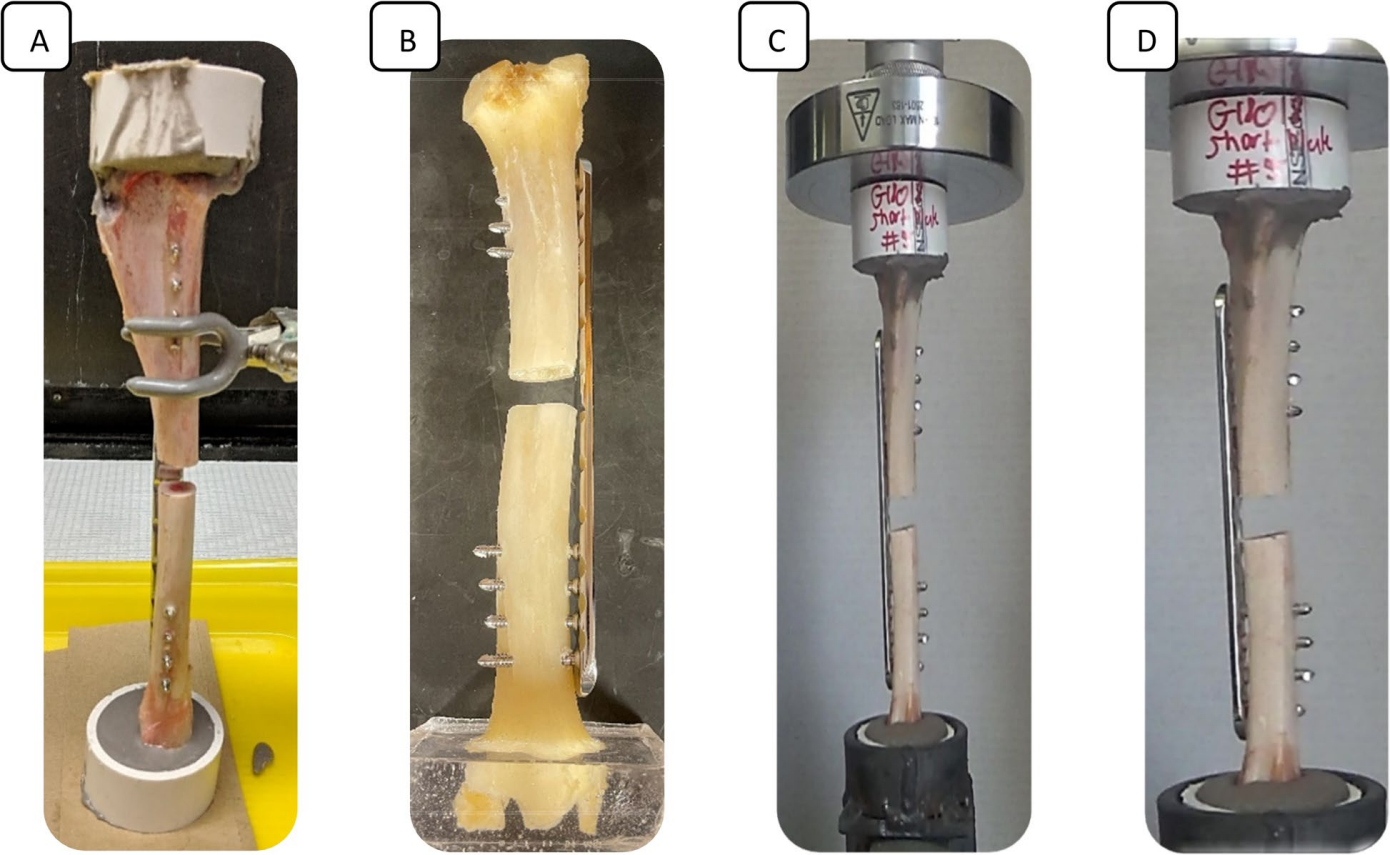
**A** Lateral view of tibia specimen mounted in poly-methyl-methacrylate prior to mechanical testing. **B** Caudal view of tibia specimen utilized for model illustration. **C** and **D** Cranial and lateral views of tibia specimens mounted in mechanical testing system prior to compression testing. In all specimens, the 1 cm segmental ostectomy is stabilized using an 18 cm (**A** and **B**) or 14 cm (**C** and **D**) 316 stainless steel low contact round double threaded 8-hole, 4.5-mm thick locking plate applied in a bridging fashion with four bicortical locking screws proximal to the defect and four distal

Seventeen left hindlimbs were collected from adult goats that had died or were euthanized for reasons other than orthopedic disease. Specimens were frozen at -20 °C and thawed at room temperature (22 °C) 24 h prior to testing. Prior to surgical manipulation, limbs were randomly assigned to either long (18 cm) or short (14 cm) plate length groups. All surgeries were performed by the same investigator under the supervision of a veterinary surgeon with extensive orthopedic experience. The tibia was approached via a roughly 20 cm incision along the medial surface of the limb, extending from immediately proximal to the medial malleolus to immediately distal to the medial condyle of the tibia. Overlying soft tissue and periosteum were elevated from the bone to allow plate placement over the craniomedial mid-diaphysis. Custom-designed 316 stainless steel low contact round double threaded 8-hole, 4.5-mm thick locking plates with a solid central portion (Veterinary Orthopedic Implants, St. Augustine, FL, USA) were used for both the in vitro and in vivo experimentation. Prior to limb dissection and creation of the defect, the locking plates were secured to the tibias using eight 4.0 mm self-tapping locking screws. Perpendicular alignment was ensured during screw hole drilling using a 3.2 mm diameter locking-head drill sleeve. Screws were hand tightened until tight engagement between the screw and plate threads was achieved at a 4.0 nm torque. Once the plate was secured to the bone, the remaining soft tissue around the tibia was excised to harvest the plated tibia. Tibial length and mid-diaphyseal width and depth measurements were obtained using electronic calipers or standard tape measure and were recorded for future analysis. A 1 cm segmental ostectomy was created at the mid-diaphysis of the tibia using a diamond-blade band saw. Care was taken to avoid saw damage to the plate during ostectomy. Proximal and distal epiphyses of each specimen were embedded in poly-methyl-methacrylate (Technovit, Jorgensen Laboratories, Loveland, CO, USA) in a 5 cm diameter, 2.5 cm high cylindrical mold for axial compression testing (Fig. 1).

#### Mechanical testing

The tibia/plate constructs were assessed using a materials testing system (Instron 5965, Instron, Norwood, MA, USA) with a custom jig to ensure stable hold of the constructs during testing. Specimens were tested in single cycle axial monotonic compression until failure at a rate of 20 mm/min. End of test criteria were plate failure defined as plastic deformation (bending) of the plate such that the two bone segments touched, bone failure defined as a visual or auditory cue of fracture confirmed by a vertical yield point on the generated load-deformation curve, or arrival at maximal load for the mechanical testing system, pre-set at 4.9 kN. Data was recorded using commercially available software (Bluehill3, Instron, Norwood, MA, USA) and processed using a custom Matlab script (The MathWorks Inc., Natick, MA, USA).

### In vivo experiments

#### Goats

All study procedures were approved by the University of Tennessee Animal Care and Use Committee (protocol numbers 2741 and 2383) and adhered to the National Institute of Health’s Guide for the Care and Use of Laboratory Animals [23]. Boer-cross, adult goats (*n* = 161 females; mean weight 50.6 ± 7.58 kg, weight range 29 – 73 kg) were used in this study. Preoperatively, goats were housed in small group pens in groups of four to six (≥ 17 ft^2^ per goat); postoperatively, goats were housed individually in adjacent pens (≥ 20 ft^2^ per goat) for a minimum of seven days, followed by group housing based on goat behavior, clinician discretion, and housing availability. Flooring included a layer of wood shavings laid on top of rubber mats over concrete flooring in a conditioned housing facility for the duration of the study. The goats were fed a balanced ration of grass hay, supplemental grain mix, and alfalfa as needed based on body condition and weight change. Free choice fresh water was provided via automatic waterers in group housing and in water buckets in individual pens. Goats were weighed at study entry, weekly for the first thirty days postoperatively, and monthly for the remainder of the study. The goats enrolled in this study were part of a series of orthopedic research projects assessing bone healing following segmental tibial ostectomy. Criteria for inclusion of goats in this study included those having a segmental tibial ostectomy (2 cm defect), bridging fixation using the single, custom designed locking plate, and a minimum of two postoperative orthogonal radiographs available for review and measurement at various time intervals. Exclusion criteria included diagnosis of postoperative osteomyelitis or identification of plate bending on postoperative radiographs during the goats’ enrollment in the ongoing orthopedic research.

#### Surgery

**Fig. 2:**
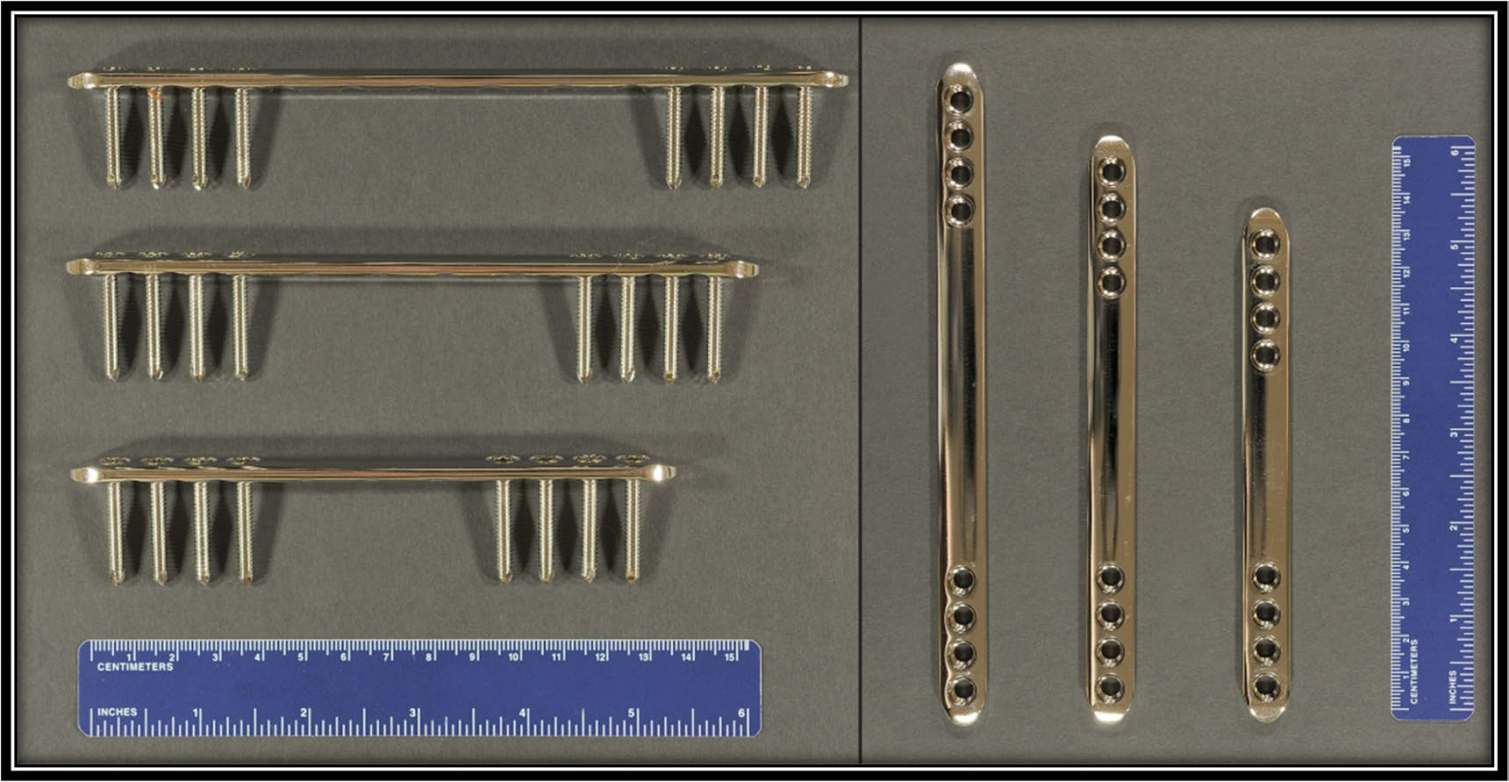
Custom-designed low contact, round screw hole, double threaded 8-hole, 4.5-mm thick locking plates. Three plate lengths were utilized: 14 cm, 16 cm, and 18 cm. In surgery, the plate was centered over the ostectomy and secured with eight 4.0-mm diameter locking-head self-tapping screws, four in the proximal tibial segment and four in the distal tibial segment. Fixation working length directly reflected the plate length selected

A mid-diaphyseal 2.0 cm segmental ostectomy was performed on the right hind tibia of each goat. The tibia was stabilized using a custom-designed low contact, round locking screw hole, double threaded 8-hole, 4.5-mm thick locking buttress plate with a solid central portion between the screw holes (Veterinary Orthopedic Implants, St. Augustine, FL, USA). Three options for plate length (14 cm, 16 cm, and 18 cm; Fig. 2) were available for use and plate selection was made at the time of surgery based on surgeon assessment of the tibia. The plate was centered over the ostectomy and secured with eight 4.0-mm diameter self-tapping locking screws (Veterinary Orthopedic Implants, St. Augustine, FL, USA), four in the proximal tibial segment and four in the distal tibial segment. Surgical procedures were conducted as previously described [4]. Postoperatively, goats were maintained in full limb bandages with medial and lateral plastic splints (Premier1Supplies, Washington, IA, USA) that spanned the limb from foot to stifle. Bandage changes occurred every two days for the first two weeks, and then were discontinued between 2 and 4 weeks postoperative based on clinical condition and at the discretion of the supervising veterinarian.

Radiographic examination of the tibia, consisting of a minimum of two orthogonal views centered at mid-tibia, were performed immediately postoperatively, one day following surgery, and then at monthly or bi-monthly intervals as directed by the ongoing orthopedic research protocol. Computed tomographic examination of the operated limb was performed at the time of plate removal, either postmortem or under general anesthesia as dictated by the experimental design. Goats were humanely euthanized at predetermined timepoints of 3, 6, 9, or 12 months postoperatively.

Goats were categorized into two outcome categories, fracture morbidity and no fracture morbidity, based on medical record review and radiographic confirmation of any complete or incomplete tibial fracture. Cases with minor fragmentation of the trans-cortex at the region of bi-cortical screw engagement were not classified as fractures for this analysis.

### Plate position analysis

#### Proximodistal positioning and tibial coverage

**Fig. 3:**
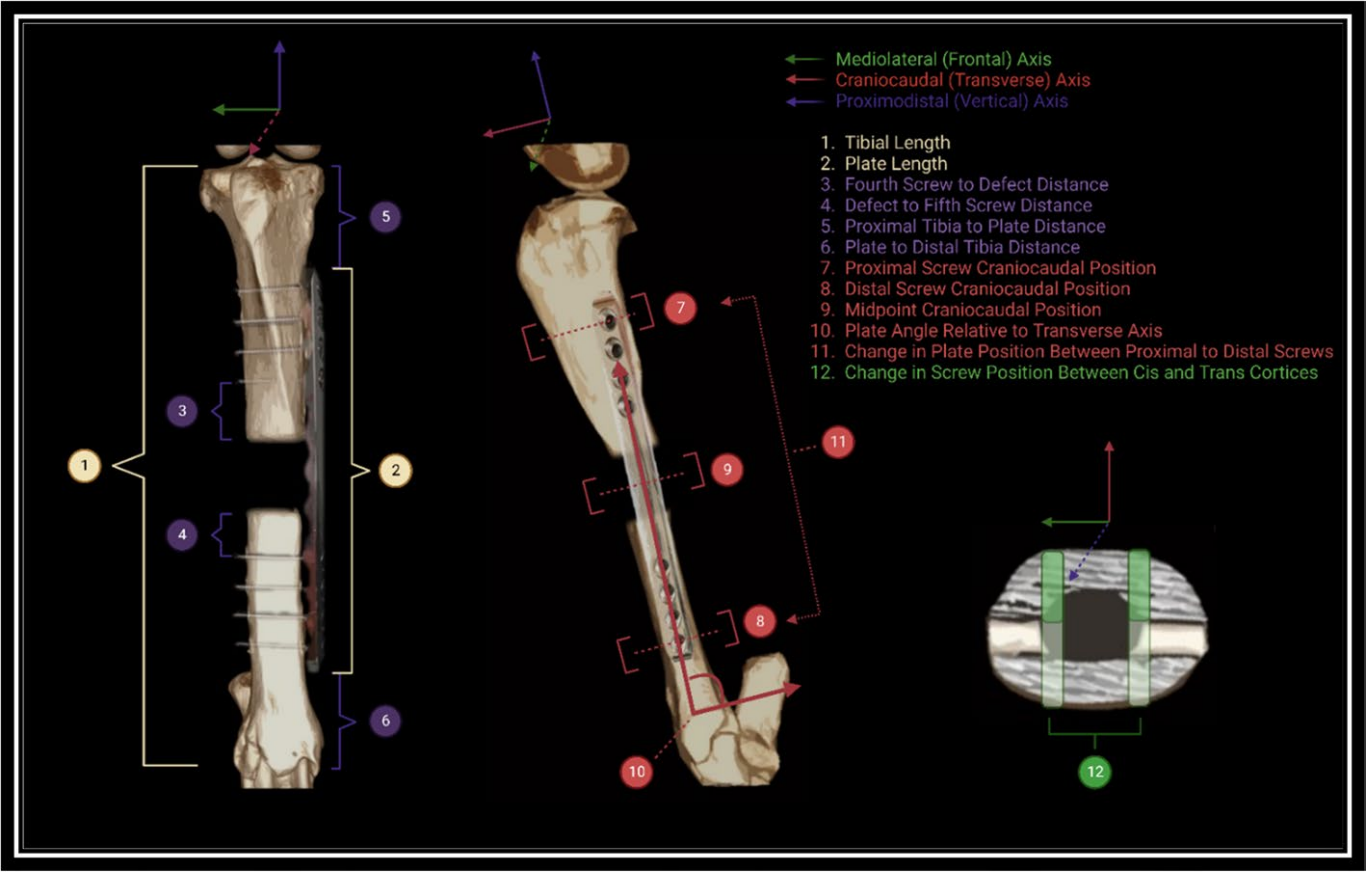
Diagram of tibial axes and measurements included in plate characteristics and positioning variables. Distances were measured in millimeters from calibrated radiographic or computed tomographic data. Positions were calculated as ratios of calibrated distances, and angles were calculated from calibrated distances and known constants. Image created using Biorender.com

Proximodistal position, tibial length, and relative tibial coverage were assessed using postoperative craniocaudal and lateromedial radiographic views (Fig. 3). Plate lengths were either 14 cm, 16 cm, or 18 cm, and these known distances were used to calibrate the images for further measurements. Screws were numbered proximally to distally with “1” as the most proximal screw and “8” as the most distal screw. Recorded variables included measured tibial length, distance from proximal tibia to proximal extent of locking plate (PrT-Pl), distance from distal tibia to distal extent of locking plate (Pl-DT), distance from the fourth screw to defect (S4-D), and distance from defect to fifth screw (D-S5). The tibial medial condyle served as a repeatable landmark for the proximal extent of the tibia and the medial malleolus was used as the distal extent. With the exception of S4-D and D-S5, all measurements were performed in duplicate utilizing the craniocaudal and lateromedial views, and respective averages were utilized for calculations and statistical analysis. S4-D and D-S5 measurements were performed utilizing the craniocaudal view only, and raw data was statistically analyzed. Relative tibial coverage ratios (TCR) were calculated using the following equation:

1 $$Tibial\;Coverage\;Ratio=\frac{\mathrm{Plate}\;\mathrm{Length}}{\mathrm{Measured}\;\mathrm{Tibial}\;\mathrm{Length}}$$

### Craniocaudal positioning

Craniocaudal (anterior–posterior, AP) positioning was assessed using computed tomographic examinations at the time of plate removal with the screw tracks serving as positional markers (Fig. 3). Craniocaudal positions of the plate at the proximal and distal tibia were assessed using the most proximal (S1) and most distal screw tracks (S8), and position was expressed as a ratio using the following equation:

2 $$Position=\frac{\mathrm{CrS}}{\mathrm{TD}}$$

where CrS represents the distance from the cranial tibia to cranial extent of screw track and TD represents tibial depth at screw midpoint.

Craniocaudal position of the plate at its midpoint (Mid-AP) was calculated as the linear mean of the proximal screw craniocaudal position (Pr-AP) and the distal screw craniocaudal position (D-AP), and the change in relative plate position between the proximal and distal tibia (PrD-Diff) was expressed as the difference between Pr-AP and D-AP. The angle of plate position relative to tibial transverse axis (AP-Angle) was calculated using the following equation:

3 $$AP-Angle = {\mathrm{cos}}^{-1}\left(\frac{{CrS}_{P} - {CrS}_{D}}{Z}\right)$$

where ${CrS}_{P}$ represents the distance from cranial tibia to cranial extent of screw track at the most proximal screw and ${CrS}_{D}$ represents the same measurement at the most distal screw track. Z designates the known distance between the first and last screw holes on either the 14 cm, 16 cm, or 18 cm plates and served as a conditional constant based on plate length group.

### Mediolateral angular positioning

Mediolateral angular positioning, in terms of biocentric deviation from the mediolateral plane, was assessed using computed tomographic examinations at the time of plate removal with the screw tracks serving as positional markers (Fig. 3). All plates were applied in a craniomedial to caudolateral fashion at the time of surgery. To estimate the magnitude of deviation from the tibial frontal axis, the change in relative plate position between the medial (cis) cortex and lateral (trans) cortex (ML-Diff) was calculated using the following equation:

4 $$ML-Diff = \frac{{CrS}_{L}}{{TD}_{L}} - \frac{{CrS}_{M}}{{TD}_{L}}$$

where ${CrS}_{L}$ represents the distance from the cranial tibia to cranial extent of screw track immediately adjacent to the lateral (trans) cortex and ${CrS}_{M}$ represents the distance from the cranial tibia to cranial extent of screw track immediately adjacent to the medial (cis) cortex. ${TD}_{L}$ and ${TD}_{M}$ represents tibial depth at the selected lateral and medial points.

#### Statistical analysis

Mechanical testing variables, in vitro bone measurements, and in vivo sample characteristics such as body weight distribution were examined using multivariate analysis of variance (MANOVA) and univariate analysis of variance (ANOVA) with treatment as the independent variable. Diagnostic analyses were conducted on residuals for normality model assumption using Shapiro–Wilk test. Post hoc multiple comparisons were performed with Tukey’s adjustment. Data were presented as mean ± SD.

The effects of plate positioning and length on fracture status were evaluated using logistic regression. To detect multicollinearity issues, the variables of goat intake body weight, tibial length, selected plate length, plate positioning, and tibial coverage were assessed for correlation using Pearson’s product moment correlation coefficients and variables expressing significant correlation were limited in final analysis. Then, the effects of plate position, length, and relative tibial coverage on fracture status were screened using multivariate logistic regression analysis controlling for known plate length with the fracture status as the binary response variable. The effects of craniocaudal and mediolateral position on fracture status were analyzed using multivariate logistic regression analysis with incidence of fracture as the binary response variable. Qualitative data are presented as percentages and count numbers. Statistical significance was identified at the level of 0.05. Analyses were conducted in SAS 9.4 TS1M6 for Windows 64x (SAS institute Inc., Cary, NC, USA) and IBM SPSS Statistics v. 28 (IBM Corp. Armonk, NY, USA).

## Results

### In vitro mechanical testing

A total of seventeen tibias were utilized in this experiment. Each tibia was randomly assigned to either long (18 cm, *n* = 9) or short (14 cm, *n* = 8) plate length groups. Tibial characteristics are presented in Table 1. Tibial length and diaphyseal depth did not differ significantly between groups (*p* = 0.753 and *p* = 0.282 respectively), but the long plate group had statistically significantly larger mean diaphyseal width than the short plate group (2.0 cm and 1.8 cm respectively, *p* = 0.005). As per in vitro study design, tibial coverage ratios differed between groups with significantly more tibial diaphysis spanned by the plate in the long plate group (*p* < 0.001).

**Table 1:** Results of in vitro plate length analysis. Despite significant differences in tibia coverage ratio, no significant differences in maximum load or total strain were detected during axial compression to failure

Classification	Variable name	Unit	Plate length		*P* Value
			**14 cm**	**18 cm**	
			*(Mean* ± *St. Dev.)*	*(Mean* ± *St. Dev.)*	
Tibial Characteristics	Tibial Length	cm	24.0 ± 1.3	24.0 ± 1.6	0.753
	Tibial Diaphyseal Width (Mediolateral)	cm	1.8 ± 0.14	2.0 ± 0.12	**0.005**
	Tibial Diaphyseal Depth (Craniocaudal)	cm	1.6 ± 0.11	1.6 ± 0.14	0.282
Plate Characteristics	Tibial Coverage Ratio	N/A (ratio)	0.591 ± 0.032	0.754 ± 0.052	** < 0.001**
Biomechanical Testing	Maximum Load	Newtons	4314 ± 700	4396 ± 874	0.793
	Total Strain	mm	7.46 ± 2.48	7.56 ± 2.99	0.668

Fourteen of seventeen tibia-plate constructs failed during axial compression testing limited to 4.9 kN (Table 2). Of these, four failed by bone fracture (2 short plate and 2 long plate constructs), six failed by plate bending to the point of impingement of the opposite cortex (4 short plate and 2 long plate constructs), and four failed by initial bending followed by acute bony fracture (2 short plate and 2 long plate constructs). Three constructs, all of which were long plate constructs, reached the test endpoint without apparent failure; these constructs were included in statistical analysis with the measured load and strain at time of test endpoint utilized as maximum load and total strain.

**Table 2:** Outcomes and modes of failure for in vitro plate-bone construct mechanical testing. Fourteen of seventeen constructs failed by either bone fracture, plate bending to the point of bone impingement at the opposite cortex, or initial plate bending followed by fracture. Three long plate constructs reached the 4.9 kN test endpoint without failure

Outcome	Mode of failure	Number of constructs	
		**14 cm**	**18 cm**
		*n* = 8	*n* = 9
**Failure**	Fracture	2	2
	Plate Bending	4	2
	Plate Bending, then Fracture	2	2
**Non-Failure**	N/A	0	3
*4.9 kN test endpoint*			

**Fig. 4:**
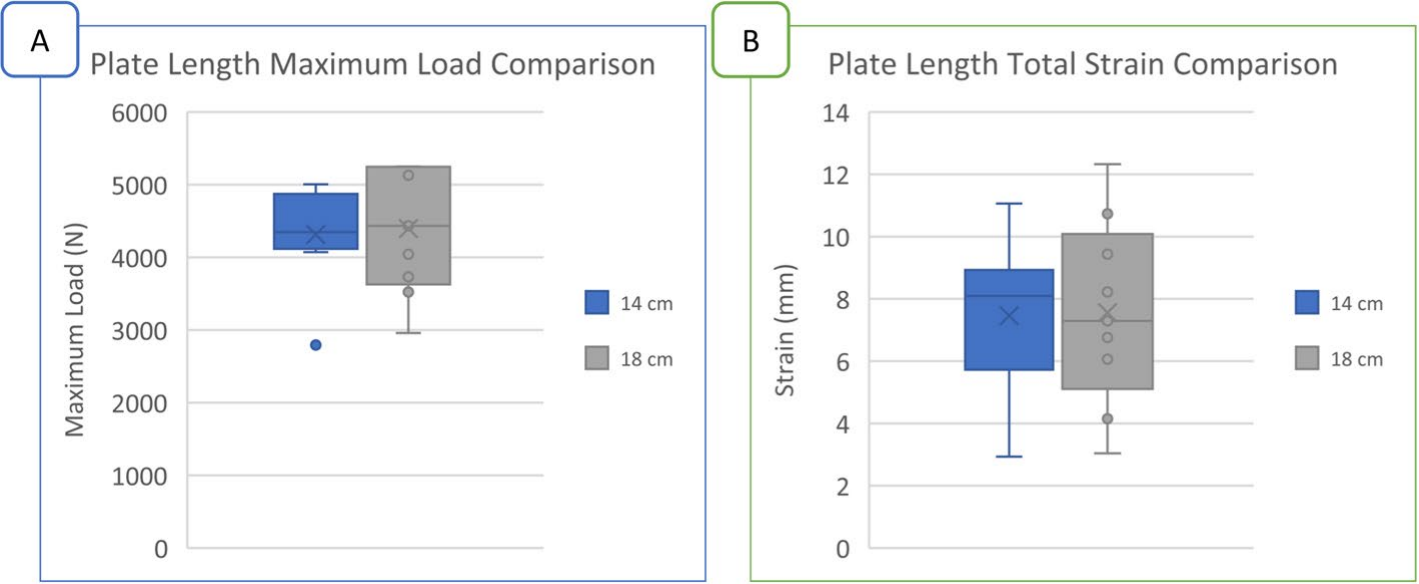
Box-and-whisker plots of **A**) maximum load (N) and **B**) total strain (mm) during compression to failure as a function of plate length. No significant differences were detected between 14 and 18 cm plates in vitro

Mean maximum loads for the short and long plate groups were 4314 ± 700 N and 4396 ± 874 N, respectively, and total strains were 7.46 ± 2.48 mm and 7.56 ± 2.99 mm, respectively (Fig. 4). No significant differences in compressive maximum load or total strain were observed between plate length conditions (*p* = 0.793 and *p* = 0.668, respectively).

### Clinical morbidity

**Fig. 5:**
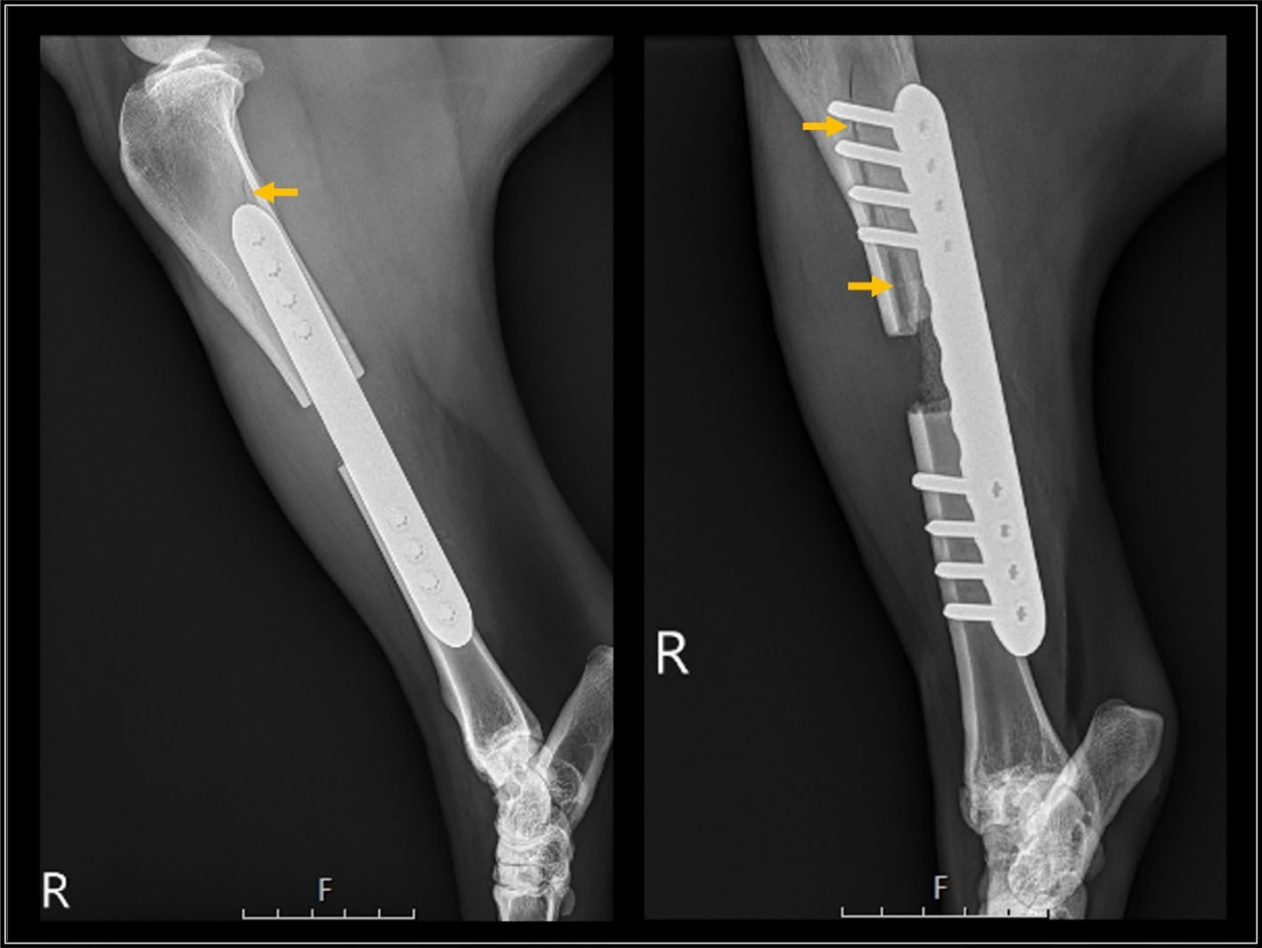
Lateromedial and caudolateral-craniomedial oblique radiographic projections of fractured caprine tibia (yellow arrows). These images illustrate the most common fracture configuration, an incomplete, longitudinal oblique fracture of the proximal tibial segment with the fracture line following the screw holes

Of 161 goats, 133 met the inclusion criteria for this study. Radiographic studies utilized for plate position measurements and analysis were acquired at a median of 1 day postoperatively (range 1 – 2 days). Thirty goats received the 14 cm plate, 38 received the 16 cm plate, and 65 received the 18 cm plate. On initial correlation analysis, surgeon-selected plate length was significantly associated with both the body weight of the goat and the measured radiographic tibial length (*p* = 0.043 and *p* < 0.001, respectively). However, goat body weight and measured tibial length were not significantly correlated (*p* = 0.322), and body weights of goats with and without fracture were not significantly different (*p* = 0.236). Due to these correlations that cause multicollinearity issues in logistic analysis, only the variable of plate length was included in further analysis for association with postoperative fracture complications. Of the 133 goats, 22 (16.5%) experienced a fracture complication during their assigned postoperative period ranging from 3 to 12 months. Fracture incidence as a function of plate length was 56.7% (17/30) for 14 cm plates, 7.9% (3/38) for 16 cm plates, and 3.1% (2/65) for 18 cm plates. The most common fracture configuration noted was a longitudinal oblique fracture of the proximal tibial segment with the fracture line typically following the screw holes proximally (Fig. 5).

Plate length was significantly associated with postoperative fracture complication (Table 3) with greatest fracture incidence noted in the 14 cm group (*p* < 0.0001). Exploring further, calculated tibial coverage ratios were significantly less in goats that suffered a postoperative fracture compared to those without (0.672 ± 0.039 and 0.748 ± 0.039 respectively, *p* < 0.001). Analysis of measured proximodistal, craniocaudal, and mediolateral positioning variables is presented in Table 4. Of these variables, only the proximodistal distance from the gap defect to the fifth screw, the closest screw to the gap within the distal segment, was significantly associated with fracture. Goats that experienced a fracture complication had a mean distance of 21.4 ± 8.34 mm between the defect and fifth screw whereas goats without fracture had a mean distance of 28.5 ± 7.67 mm (*p* = 0.008). No craniocaudal positioning variables or mediolateral deviation from the bicentric plane were significantly associated with the development of postoperative fracture complications.

**Table 3:** Selected plate lengths for all goats included in retrospective radiographic review. Plate length was significantly associated with fracture complication (*p* < 0.001) with increased fracture incidence in the 14 cm plate group

Plate selection		Outcome		*P*-Value
Plate length (cm)	N	No fracture	Fracture	
14	30	13	17	
16	38	35	3	
18	65	63	2	
**Total**	**133**	**111**	**22**	** < 0.0001**

**Table 4:** Plate characteristics and positioning variables identified on radiographic and computed tomographic examination and their association with fracture complication outcome

Classification	Variable name	Variable description	Unit	Outcome		*P*-Value
				**No fracture**	**Fracture**	
				***(Mean* ± *St. Dev.)***	***(Mean* ± *St. Dev.)***	
Plate Characteristics	TCR	Tibial coverage ratio	N/A (ratio)	0.748 ± 0.047	0.682 ± 0.039	** < 0.001**
Proximodistal Positioning	PrT-Pl	Distance from proximal tibia to proximal extent of plate	mm	26.30 ± 9.89	35.51 ± 7.74	0.201
	Pl-DT	Distance from plate to distal tibia	mm	30.89 ± 5.69	32.66 ± 7.86	0.535
	S4-D	Distance from fourth screw to defect	mm	28.64 ± 8.91	18.69 ± 5.69	0.219
	D-S5	Distance from defect to fifth screw	mm	28.53 ± 7.67	21.39 ± 8.34	**0.008**
Craniocaudal Positioning	Pr-AP	CrCd plate position at proximal tibia	N/A (ratio)	0.667 ± 0.085	0.659 ± 0.047	0.228
	D-AP	CrCd plate position at distal tibia	N/A (ratio)	0.436 ± 0.079	0.399 ± 0.067	0.32
	Mid-AP	CrCd plate position at mid-tibia	N/A (ratio)	0.552 ± 0.056	0.529 ± 0.041	N/A
	AP-Angle	Angle of plate position relative to tibial transverse axis	Degrees	84.63 ± 1.44	84.15 ± 1.37	0.221
	PrD-Diff	Change in relative plate position between proximal and distal tibia	N/A	0.231 ± 0.120	0.260 ± 0.082	N/A
Mediolateral Positioning	ML-Diff	Change in relative plate position between medial (cis) and lateral (trans) cortices	N/A	0.052 ± 0.048	0.052 ± 0.048	0.825

## Discussion

This study aimed to assess the effects of surgeon-selected factors including locking plate length, plate positioning, and relative extent of tibial coverage on fixation failure, in the form of postoperative fracture. The results highlight the importance of caution when translating in vitro modeling to in vivo application of orthopedic techniques. Mechanical testing of two locking plate constructs of varying length and tibial coverage showed no difference in mechanical strength or construct stability when exposed to single cycle to failure compressive force in vitro, but upon in vivo application of the construct, significant associations of plate length and tibial coverage with construct failure, in the form of postoperative fracture, were noted. The locking plate and locking compression plate combine mechanical stability of a type-1 external fixator with the benefits of internal fixation such as lower infection risk and lack of external interference [30]. Mechanical testing of LCP stabilized gap defects in vitro have documented superior strength against bending and compressive forces in vitro when compared to conventional dynamic compression plates, but questions have been raised regarding the fixation’s torsional strength [1–3, 14, 15, 21]. In a biomechanical study of tibiofemoral contact forces in sheep, Taylor et al. documented significant axial force conveyed to the tibia from the tibiofemoral joint, but unlike humans, sheep experience a greater magnitude of craniocaudal and mediolateral sheer force originating at the stifle [31]. The authors hypothesized that these forces were caused by the obliquity of muscular forces acting on the joint due to a comparatively wide range of motion, and these forces translate to significant off-planar sheer and torsional stresses exerted on the ovine tibia [31]. Similar biomechanical analysis of caprine tibiofemoral forces and tibial stresses has not been conducted, but similarities in body mass, gait, and relative musculoskeletal size/segment lengths allow for crossover comparison. While axial compressive mechanical testing adequately replicates human tibial stresses associated with upright gait, the model is limited in translation to the unique biomechanics of a goat hind limb. In this context, we suspect that the differences in LP construct stability between in vitro modeling and in vivo implementation may be attributed to the secondary sheer and torsional stresses exerted on the tibia during gait, as opposed to mere compressive overloading. The in vitro model specifically explored the effects of plate length on single-cycle compressive load to failure. In vivo, the plates were exposed to not only cyclic compressive loading through gait, but also to stresses associated with normal recumbency, social behaviors, and other multiplanar, complex motions, thus accounting for the disparity between in vitro and in vivo outcomes in this study. Further biomechanical studies, both in vitro and in vivo, are necessary to refine our models and explore the mechanical forces acting on this fixation commonly used in translational orthopedic research.

As noted above, no significant differences in compressive maximal load or total strain were observed during compressive mechanical testing of two LP constructs of markedly different plate lengths. This differs from several in vitro models that noted a direct relationship between LP construct rigidity and plate length [5, 27, 33]. In a mechanical study utilizing simulated human supracondylar femoral fractures, locking plate length was significantly associated with construct stiffness, and longer plates generated significantly stiffer constructs regardless of number of screws and working length [33]. Cao et al. utilized finite element analysis modeling of human tibial fractures to test numerous surgeon-controlled fixation elements, including plate length, positioning, and material; they noted that construct rigidity increased with plate length under medial plating conditions and suggested that appropriate plate length selection is vital to reducing the risk of fixation failure [5]. The current study also evaluated varying working lengths of fixation, due to the custom nature of the locking plates utilized. Similar to previous mechanical studies, no significant effect of working length on construct stability was noted during compressive testing to failure [27, 33]. However, one study noted differences in yield load, specifically, between constructs of short and long working lengths, and shorter working lengths were significantly associated with greater maximum load but not overall construct stiffness during cyclic compressive testing followed immediately by compressive load to failure [6].

One limitation of the current study is the utilization of only one mode of in vitro mechanical testing, compressive load to failure. Due to restrictions in sample size and specimen availability during this experiment, further modes of loading such as bending or torsion and further test conditions such as cyclic loading were not conducted; therefore, interpretation of in vitro mechanical data is limited to ultimate compressive strength and stability, and we acknowledge the limitations of translation of this simplified model into clinical practice. Similarly, ostectomy lengths differed between the in vitro and in vivo portions of this project (1 cm versus 2 cm). The in vitro mechanical testing aimed to answer the question, “Do you lose fixation compressive strength when you use a shorter plate in this model?” Thus, the bridging nature of fixations of different lengths was the focus, and a 1 cm defect allowed inclusion of a bending endpoint (trans cortex-to-cortex contact) for comparison. On the other hand, the in vivo portion of this experiment utilized a 2 cm ostectomy as a standardized delayed union model of bone healing. The difference in ostectomy lengths reflects each respective project’s goals, but we acknowledge that it limits direct translation of in vitro to in vivo. Further mechanical studies evaluating caprine tibial gap defect locking plate fixations are recommended to evaluate the effects of plate length, working length, and ostectomy length under additional loading conditions. Finally, following random allocation of caprine tibias into either the long or short plate fixation groups, a significant difference in tibial diaphyseal width was noted. However, no corroborating differences in tibial length or diaphyseal depth were appreciated, and the difference, while significant, represents only two millimeters. Therefore, while this group difference could, in theory, affect the results of mechanical testing, we suggest that its clinical relevance is limited and does not represent a major limitation of the mechanical tests.

The current study retrospectively assessed certain surgeon-selected factors such as plate length and plate positioning during locking plate stabilization of a 2 cm caprine tibial segmental defect in vivo. To our knowledge, this is the first publication to explore these factors in small ruminants (sheep and goats) and few publications regarding long-term outcome of long-bone locking plate fixation exist in the veterinary literature [18]. In human medicine, locking compression plate fixation is commonly applied to supracondylar femoral fractures, and retrospective analysis of patient and surgeon-related factors related to fixation failure has associated shorter plate lengths with increased incidence of implant failure and fracture [26]. In one retrospective analysis of locking plate fixation of human femoral fractures (*n* = 335 fractures), Ricci et al. concluded that constructs with a plate length corresponding to 9 holes or greater were significantly less likely to fail than shorter constructs, as associated with the possibility of stress riser formation with the application of plates with lesser femoral coverage [26]. In the current study, both plate length, alone, and calculated tibial coverage ratios were significantly associated with risk of postoperative fracture. In this goat model, cases without fracture complications had plates covering roughly 75% of the tibia as opposed to 65–67% coverage in fracture cases. The results of this study suggest that the maximal plate length possible, based on anatomic features and fracture configuration, be used when applying locking plates for the purpose of stabilizing non-load sharing fractures, and follow-up studies exploring additional plate lengths will allow for further elucidation of the optimal coverage ratio for this fixation.

In contrast to plate length and relative tibial coverage, plate positioning, as described by radiologically measured proximodistal, craniocaudal, and mediolateral variables, was not significantly associated with fracture complications with one exception, the distance between the gap margin and the fifth screw (first screw in distal tibia). In fracture cases, the distance between the gap margin and fifth screw was significantly shorter (by roughly 7 mm) than that in cases without fracture. Utilizing a two-dimensional finite element analysis model of bridging locking plate fixation of a simple transverse diaphyseal human femoral fracture, Giordano et al. demonstrated that the highest concentration of stress occurred around the screws closest to the fracture margin and suggested that a threshold of distance between the fracture line and closest screws is necessary to allow for safe deflection of stress and overall construct stability [10]. This provides a possible explanation for the significant association between shorter distance from the defect to fifth screw and postoperative fracture complication observed in the current study.

Plate working length is defined as the distance between the two screws closest to the fracture or gap margin [12]. In the current study, custom locking plates of three lengths were utilized, and working length was fixed for each of the plate length categories; thus, experimental working lengths directly reflect the plate lengths selected, representing absolute correlation, and only plate length variables were included in the final logistic regression analysis. Due to the retrospective nature of the study, we are unable to differentiate between the effects of overall plate length and the effects of fixation working length on fracture outcomes, and this represents a major limitation of the study. Previous in vitro and in vivo analyses of the effects of working length on locking plate fixation have yielded mixed results [6, 10, 12, 33]. Using in vitro FEA modeling, Giordano et al. described a direct correlation between increasing working length and construct stability through effective deflection of stress, but in mechanical testing of various working lengths for LCP fixation of canine femoral gap defects, Chao et al. described significantly higher strength (yield load) in short compared to long working length constructs [6, 10]. In retrospective analyses of in vivo factors associated with distal femoral LCP fixation failure in humans, working length was not significantly associated with implant failure or with nonunion [12, 26]. In addition, in a retrospective analysis of locking plate fixation stiffness as a function of working length, neither fixation working length nor the ratio of working length to plate length were correlated with overall fixation stiffness or with complications such as delayed union or nonunion [24]. In the current study, working length was directly reflective of the selected plate length, and both short plate length and lower tibial coverage ratios were significantly associated with fixation failure. Follow-up experimentation employing either consistent plate length and variations of working length or vice versa are necessary to differentiate the effects of plate length and fixation working length in locking plate fixation of caprine tibial gap defects.

Due to the retrospective nature of this study, certain limitations in group sample sizes and plate positioning are inherent. Per the ongoing orthopedic research protocol, all plates were placed on the craniomedial surface of the tibia to allow for adequate surgical site exposure and an overall uniformity of fixation. However, the exploration of plate positioning was therefore limited to craniomedially placed plates, and the lack of data from other tibial surfaces serves as a limitation of the present study. In addition, differences in group sample sizes with relative overrepresentation of 18 cm plate length are inherent in this retrospective, convenience sample. As noted above, plate length selection was carried out by the attending surgeon at the time of gap fixation, based on evaluation of the surgical site. Thus, reported correlations of goat weight and tibial measured length with selected plate length are logical, and we suggest that these conditions reasonably simulate a clinical setting. However, conclusions regarding potential confounding effects of body weight, tibial length or size, and plate length selection cannot be made from this study. In the current study, the additional variable of tibial coverage ratio was included to link the factors of surgeon-selected plate length and goat-specific tibial size, and lesser degrees of tibial coverage, regardless of absolute tibial length, were significantly associated with postoperative fracture. In this model, goats immediately bear weight on the operated limb and though their activity is limited to stall rest, behaviors such as pacing and standing on their hindlimbs were unrestricted. Thus, quantification of stresses to the construct is goat-specific and reliant on individual behavior. Further research into the association of veterinary patient demographic factors such as age, weight, and bone dimensions, surgeon-selected factors, goat behaviors and activity levels, and orthopedic complications is warranted.

## Conclusion

Although in vitro modeling of variable plate length in relation to tibial length suggested that similar failure modes and rates could be anticipated, in vivo application revealed that both plate length and tibial coverage ratio were significantly associated with the incidence of postoperative fracture complications under the condition of segmental defects in which the bone does not contribute to load sharing. Interestingly, decreasing distance between the gap defect and the first screw distal to the gap was associated with increased incidence of fracture. Based on the results of this study, optimization of plate-to-tibia coverage through maximization of plate length may lessen the risk of postoperative fracture morbidity following locking plate fixation of diaphyseal gap defects.

## Data Availability

In accordance with institutional policy for patients’ medical records, data used in this study are not open to public but will be available upon motivated request to the corresponding author for the purpose of scientific research.

## Abbreviations

DCP: Dynamic compression plates
LC-DCP: Limited-contact dynamic compression plates
PC-Fix: Point-contact fixators
LCP: Locking compression plates
LP: Locking plates
FEA: Finite element analysis
